# Nutrient Intake Patterns Predict Homeostatic Dysregulation in an Older Quebec Population

**DOI:** 10.1093/geroni/igaa057.3101

**Published:** 2020-12-16

**Authors:** Alan Cohen, Alistair Senior, Véronique Legault, David Raubenheimer, Stephen Simpson, Nancy Presse, Pierrette Gaudreau, David Le Couteur

**Affiliations:** 1 Universite de Sherbrooke, St-Denis-de-Brompton, Quebec, Canada; 2 University of Sydney, Camperdown, New South Wales, Australia; 3 Université de Sherbrooke, Sherbrooke, Quebec, Canada; 4 University of Sydney, Sydney, New South Wales, Australia; 5 University of Sherbrooke, Sherbrooke, Quebec, Canada; 6 University of Montreal, Montreal, Quebec, Canada

## Abstract

The geometric framework for nutrition has largely been applied to macronutrients in experimental settings. Here, we utilize the framework to examine both macro and micronutrient intake patterns in observational human data. We used nutritional intake patterns (3x 24h recall per visit) of 1754 older Quebecers from the NuAge cohort to predict multi-system homeostatic dysregulation scores calculated from 30 biomarkers. Intermediate intake of both macro- and micronutrients was generally associated with lower dysregulation scores (i.e., better health). Furthermore, there were often nutrient-nutrient interactions, such that the optimal level of one nutrient depends on the intake level of others. However, higher protein intake was generally associated with better health, and results varied substantially across different dysregulation systems. Accordingly, even though nutrition does have important effects on health trajectories during aging, it will be challenging to arrive at population-level recommendations to fine-tune nutrient intake patterns to optimize health beyond “everything in moderation.” Part of a symposium sponsored by the Nutrition Interest Group.

